# Structural and magnetic properties of iron nanowires and iron nanoparticles fabricated through a reduction reaction

**DOI:** 10.3762/bjnano.6.167

**Published:** 2015-07-29

**Authors:** Marcin Krajewski, Wei Syuan Lin, Hong Ming Lin, Katarzyna Brzozka, Sabina Lewinska, Natalia Nedelko, Anna Slawska-Waniewska, Jolanta Borysiuk, Dariusz Wasik

**Affiliations:** 1 University of Warsaw, Faculty of Physics, Institute of Experimental Physics, Pasteura St. 5, 02-093 Warsaw, Poland; 2 Tatung University, Department of Materials Engineering, DeHui St. 104, Taipei, Taiwan, R.O.C; 3 University of Technology and Humanities in Radom, Faculty of Mechanical Engineering, Department of Physics, Krasickiego St. 54, 26-600 Radom, Poland; 4 Polish Academy of Sciences, Institute of Physics, Al. Lotnikow 32/46, 02-668 Warsaw, Poland

**Keywords:** iron nanoparticles, iron nanostructures, iron nanowires, magnetic properties, structural properties

## Abstract

The main goal of this work is to study the structural and magnetic properties of iron nanowires and iron nanoparticles, which have been fabricated in almost the same processes. The only difference in the synthesis is an application of an external magnetic field in order to form the iron nanowires. Both nanomaterials have been examined by means of transmission electron microscopy, energy dispersive X-ray spectrometry, X-ray diffractometry and Mössbauer spectrometry to determine their structures. Structural investigations confirm that obtained iron nanowires as well as nanoparticles reveal core–shell structures and they are composed of crystalline iron cores that are covered by amorphous or highly defected phases of iron and iron oxides. Magnetic properties have been measured using a vibrating sample magnetometer. The obtained values of coercivity, remanent magnetization, saturation magnetization as well as Curie temperature differ for both studied nanostructures. Higher values of magnetizations are observed for iron nanowires. At the same time, coercivity and Curie temperature are higher for iron nanoparticles.

## Introduction

Iron-based nanostructures attract the attention of a vast amount of scientists from all over the world. Somehow, this is related to the properties and abundance of iron in the environment. Adding that iron nanomaterials are relatively inexpensive, a lot of them are biocompatible and low-toxic, it makes them very interesting from an application point of view. So far, they have been applied in many biomedical applications including magnetic resonance imaging (MRI) contrast enhancements [1], direct drug delivery systems [2], hyperthermia treatment [3] as well as labelling and separation of biological materials [4]. Besides the biomedical exploitation, iron-based nanostructures can be used in the fields of data storage [5], catalysis [6], energy storage [7] and environmental remediation [8]. However, different properties are required for different applications. For instance, in the case of drug delivery systems nanostructures need to exhibit rather superparamagnetic behaviour with low coercivity. On the other hand, in the case of magnetic recording media there are needed materials with a high value of coercivity. Thus, it is very important to study different nanostructures to know their properties and to match them to the adequate applications.

Iron-based nanostructures in forms of nanowires as well as nanoparticles are supposed to be the most promising and therefore they are the most frequently studied. It is related to the iron features, which were mentioned before and also an enormous amount of lattice energy related to their high surface-to-volume ratios. Moreover, it is worth underlining that by changing the dimensions of nanostructures, it is possible to control the magnetic properties of these materials [9]. This opens the way for many research works in the field of iron nanoengineering.

Recently, it has been published plenty of articles about different studies of iron nanowires (Fe NWs) and iron nanoparticles (Fe NPs), including preparation, chemical functionalization and investigation of physical and chemical properties depending on different preparation conditions. However, it is difficult to find the publications where these nanostructures are compared directly regarding to their properties. Indeed, there are few articles where authors have reported that the magnetic nanorods (NRs) and nanowires (NWs) exhibit a higher value of magnetic moment than nanoparticles (NPs) of comparable volume [10–11]. But they have not taken into account that the compared materials have not been exactly the same in terms of their chemical compositions. Besides that, as far as we know, nobody has reported any experimental results comparing the magnetic properties of different nanostructures that have the same chemical compositions due to similar conditions during their fabrication. Therefore, this work can deliver the knowledge about the relationship between the forms of nanostructure, structural compositions and magnetic properties of iron nanowires and iron nanoparticles which have been synthesized via a simple chemical reduction of iron salt.

## Results and Discussion

At the beginning, it is worth to emphasize that the only difference in the fabrication processes of the two investigated nanostructures, iron nanowires and iron nanoparticles, is an application of an external magnetic field in the case of iron nanowires. The remaining part of synthesis has been performed under exactly the same conditions and with the same chemical reagents. Also, both nanomaterials have been stored in sealed vials under air-containing atmosphere. Thus, it is supposed that obtained nanostructures differ only from each other in their shapes, which has an influence on their magnetic properties. Details of the preparation process can be found in the ‘Experimental’ section below.

### Morphological and structural studies

**Figure 1:**
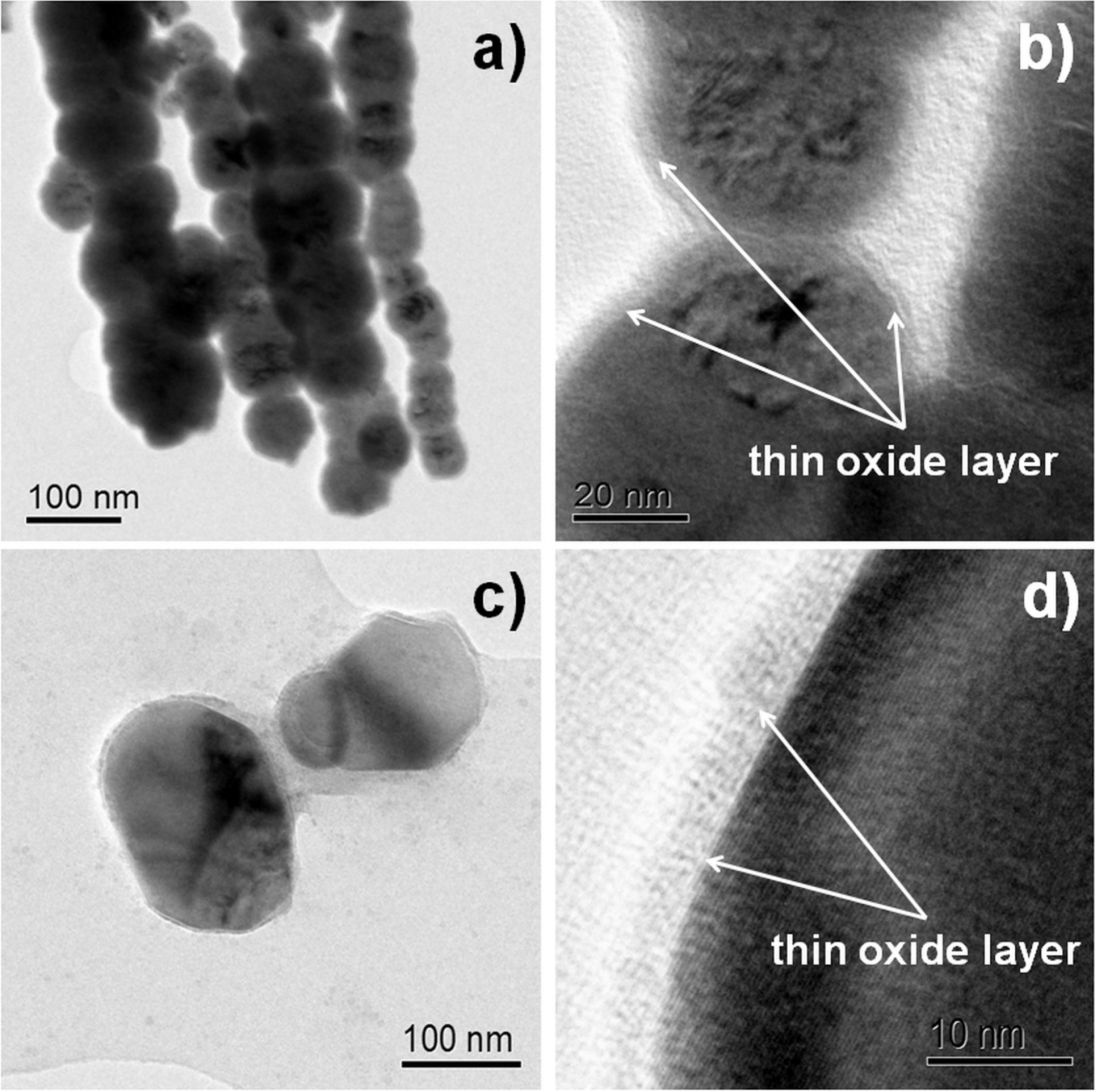
TEM images of a), b) iron nanowires and c), d) iron nanoparticles. Images b) and d) were recorded with high magnification.

The morphologies and internal structures of both studied nanostructures have been determined by means of transmission electron microscopy (TEM). Recorded images shown in Figure 1 indicate that application or absence of the external magnetic field is a crucial point for the formation of either iron nanowires or iron nanoparticles. Figure 1a demonstrates clearly that the application of the magnetic field leads to the formation of straight nanowires built from self-assembled nanoparticles, which are separated from each other by very thin layer of interface visible in Figure 1b. Similar observation has been reported previously in [12]. Also, TEM images show that iron nanoparticles are not perfect spheres. The average diameters of both Fe NWs and Fe NPs vary from 90 nm to 100 nm, while the average length of Fe NWs is equal to 5 μm. Some endings of iron nanowires and few nanoparticles have lower diameters than the average. Both investigated nanostructures are covered by a thin iron oxide layer (Figure 1b and Figure 1d) because pure iron is very reactive in the presence of even small amounts of oxygen. The thickness of this layer is around 3 nm, which is consistent with the literature [13].

**Figure 2:**
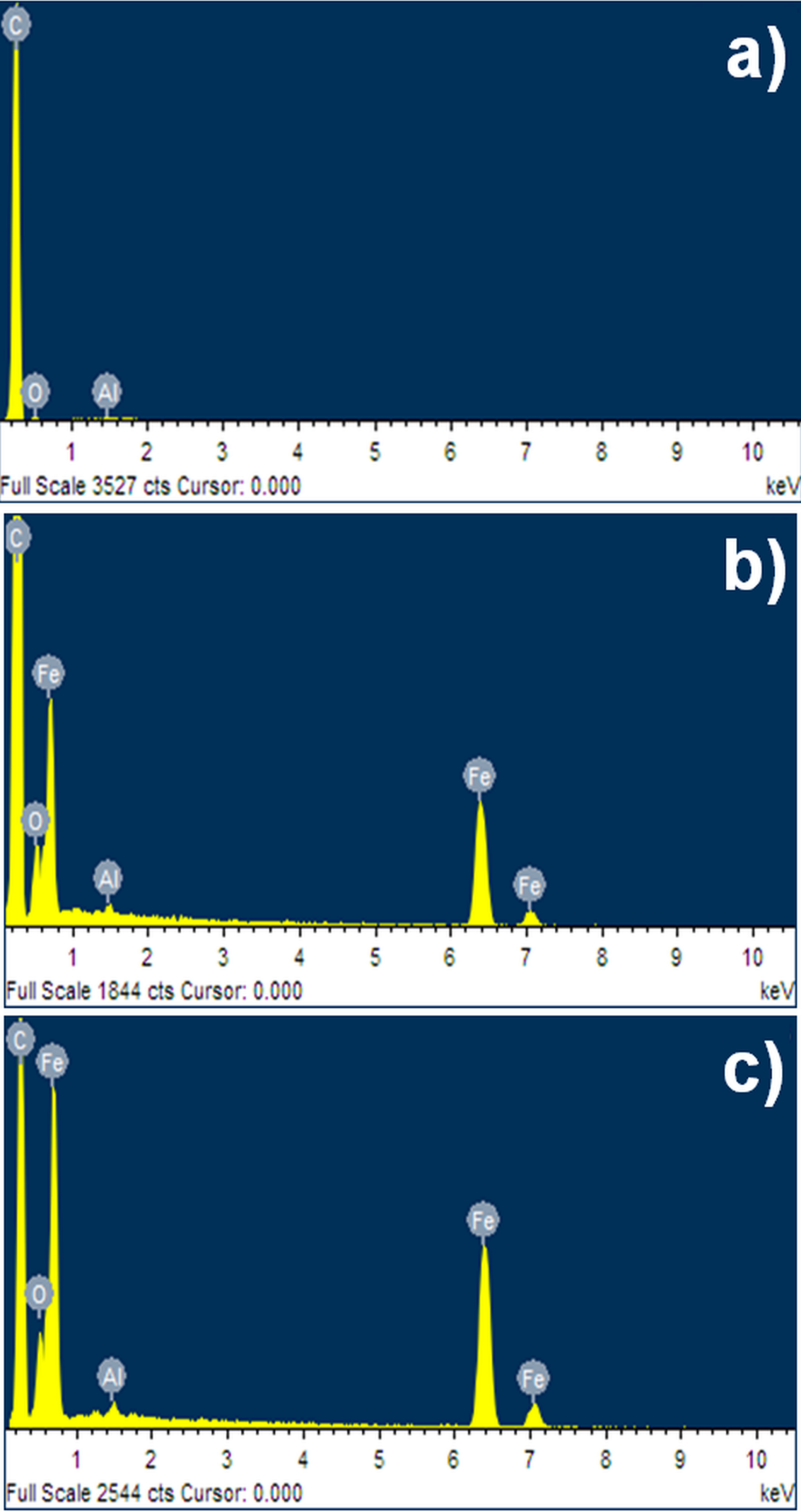
EDX spectra of the sample holder with: a) graphite cover; b) iron nanowires and c) iron nanoparticles.

Energy dispersive X-ray (EDX) spectra of empty sample holder, the holder with nanowires and the holder with nanoparticles are presented in Figure 2. All of them contain the signals originating from aluminium, carbon and oxygen. This is caused by usage of the aluminium holder, which has been covered with graphite to allow for the conduction of electrons. Moreover, it is well known that oxygen can be easily adsorbed on the surfaces of different materials. Therefore, it is visible in all of the EDX spectra. Peaks that originate from iron can be seen in Figure 2b and Figure 2c. Besides that, the intensity of the oxygen signal is increased in the case of Fe NPs. The estimated weight percentage of iron and oxygen present in iron nanowires and iron nanoparticles are summarised in Table 1. The calculations are limited to two elements (Fe and O), while all other detected elements are not taken into account. The obtained values of iron and oxygen can be explained referring to the shapes of investigated nanostructures. Iron nanoparticles have a larger surface area exposed to the atmosphere than iron nanowires. Thus, the quantity of oxygen, which has created the iron oxides on their surfaces, is slightly higher than in the case of Fe NWs.

**Table 1:** Weight percentage of iron and oxygen contents in iron nanowires and iron nanoparticles derived from EDX measurements.

	weight percentage	
	Fe	O

Fe NWs	0.79	0.21
Fe NPs	0.75	0.25

**Figure 3:**
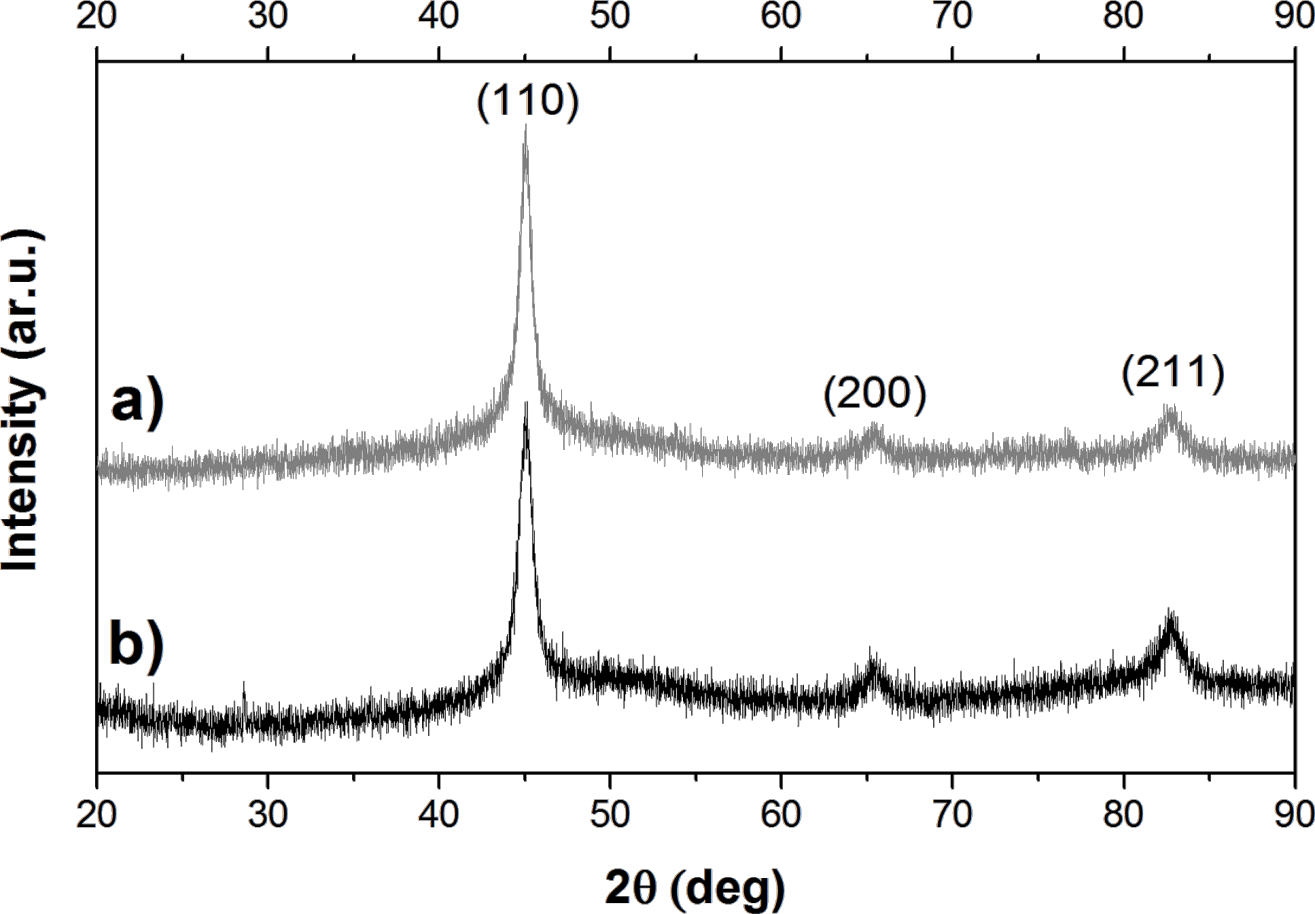
XRD patterns of a) iron nanowires and b) iron nanoparticles. Miller indices of α-Fe are given based on JCPDS no. 87-0722.

The XRD patterns (Figure 3) recorded with a step of 0.01° and a collecting time of 2 s per each step show that iron nanowires as well as iron nanoparticles are composed of the crystalline *α*-Fe phase (based on JCPDS no. 87-0722). However, the registered peaks are quite broad. This indicates that both studied nanostructures can be consisted of the small iron crystallites or they can contain a mixture of crystalline and amorphous iron phases [14]. Moreover, no signals originating from iron oxides are observed. This is in contrast to the results mentioned before. TEM and EDX measurements show clearly the presence of thin oxide layer on the surfaces of both investigated nanostructures. On the other hand, XRD is not sufficiently sensitive to determine the structures of very thin layers that are composed of several different or amorphous phases. Therefore, the transmission Mössbauer spectroscopy (TMS) has been applied for a more detailed examination of the studied nanostructures. This technique provides the information about the local chemical environment of iron ions and it is much more sensitive and precise with regard to Fe compounds than XRD.

**Figure 4:**
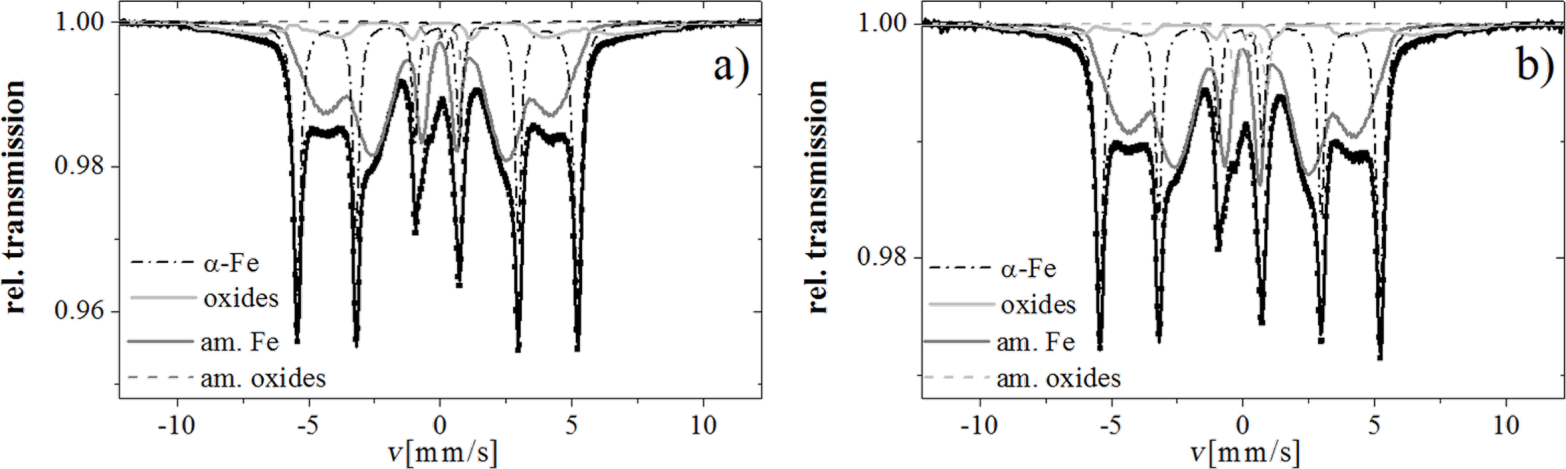
Mössbauer spectra of a) iron nanowires and b) iron nanoparticles and their main subspectra.

Mössbauer spectra of both samples are shown in Figure 4. The differences between them are hardly discernible, which reflects a similarity of the studied nanomaterials. It is also worth noting that they are quite similar to the spectrum of as-prepared iron nanowires presented in our previous studies [15]. Nevertheless, both spectra are composed of several overlapping components. The highest contribution in the spectra belongs to a broad sextet describing by the average value of the magnetic hyperfine field of about 24 T and an isomer shift of 0.03–0.04 mm/s. This component represents an amorphous iron phase with a possible contribution of oxygen atoms [16]. The second strongest signal corresponds to the presence of crystalline α-Fe with a magnetic hyperfine field of 33.05 T and an isomer shift of 0.00 mm/s. The last easily distinguishable subspectrum characterized by a high magnetic hyperfine field of over 34 T is seen in the form of side slight “wings” [15]. This component can be attributed to iron ions situated in the surface oxide layer of the nanowires. The crystal lattice, surrounding the iron ions belonging to this layer, is distorted and its symmetry is quite different than in the case of bulk iron oxides. In addition to magnetically split components, a doublet with an isomer shift of 0.41–0.43 mm/s and a singlet with an isomer shift of 0.03–0.07 mm/s and small intensity can be observed in the spectrum. Considering that in both studied materials the particles are relatively large and couple by dipolar interactions, the superparamagnetism is not very likely. Thus, the most probable origin of these contributions is related to the presence of amorphous iron oxides [17–20].

As the thickness of the oxide layer is very thin, most of its iron atoms are placed either directly next to amorphous iron or situated at the surface of nanomaterial. It has been already reported that the iron oxide layer at the surface of nanoparticles shows a tendency towards the similar crystallographic orientation as the underlying Fe core [21]. Thus, it is believed that amorphous iron, which lies between the crystalline iron core and iron oxide layer of nanowire surface, can additionally influence the creation of strongly distorted or amorphous iron oxides. The hyperfine parameters, especially the hyperfine magnetic field, related to iron ions belonging to the surface oxides, are thereby distributed. The hyperfine magnetic field values are very high, which means that the short-range order in this phase is similar as in ordered oxides, contrary to real amorphous oxides which show paramagnetic behaviour at room temperature [20,22].

The relative contribution of iron atoms belonging to the individual phases has been evaluated from the area of the corresponding subspectra and is presented in Table 2. The summaries of percentage in Table 2 rows do not always reach 100% because the components of intensity below 1% (as comparable with the measurement uncertainty) have been omitted. It is shown that the total relative intensity of subspectra representing all iron oxides is about *p*_ox_ = 10–11%, which is in a quite good agreement with the results of evaluations conducted under following assumptions: the particles are spheres with diameter *D* = 100 nm, the width of the oxides layer equals *d* = 3 nm, the Debye–Waller factor of all components of the investigated material is similar, and the weight share of iron in oxides equals *x* = 0.7:

[1]
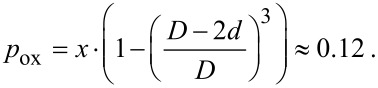


**Table 2:** The relative contribution in percent of iron atoms belonging to the individual phases derived from Mössbauer studies (the components of intensity below 1% have been omitted).

	Sextet (crystalline α-Fe)	Smeared high-field sextet (distorted oxides)	Smeared low-field sextet (amorphous iron)	Doublet (amorphous iron oxides)	Singlet (amorphous iron oxides)

Fe NWs	33.0	8.2	53.9	2.8	1.3
Fe NPs	30.6	6.1	57.2	3.6	1.7

**Figure 5:**
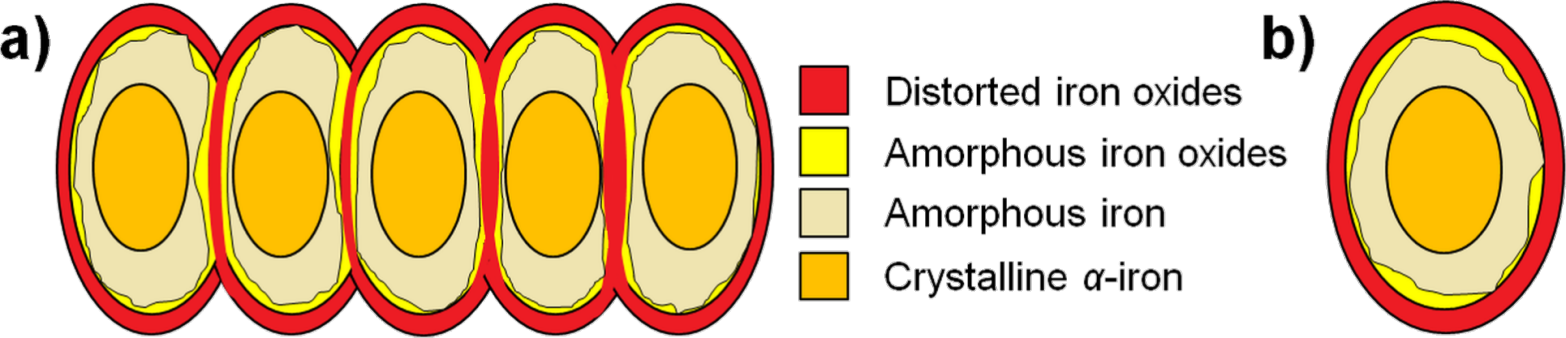
Scheme of a) iron nanowires and b) iron nanoparticles with regards to structural experimental results.

According to the experimental results, the structures of the investigated iron nanowires as well as of iron nanoparticles can be visualized as it is presented in Figure 5. The core of nanowire or nanoparticle is crystalline α-Fe. This phase of iron is covered by a quite thick layer of amorphous iron, which possibly contains some contribution of oxygen atoms. Between the amorphous iron layer and surface layer, there is a very thin layer of amorphous iron oxides. Finally, the surfaces of both studied nanomaterials are composed of the thin iron oxide films, which are distorted according to Mössbauer spectroscopy results.

### Magnetic measurements

It is well known that the properties of magnetic nanomaterials depend on several features, such as: chemical composition, shape and dimension of nano-object [9,21]. Moreover, magnetic response of nanomaterials is often a result of the material ‘history’. A lot of factors are hidden under the meaning of this word, but in the case of iron-based nanomaterials the preparation method, the storage atmosphere and side effects of radiation seem to be most important [21,23]. Therefore, in many cases it is difficult to compare the magnetic properties of different iron-based nanostructures qualitatively because their behaviours are usually related to their ‘histories’. However, in this work there are shown the results of magnetic measurements on iron nanowires and iron nanoparticles that have exactly the same ‘histories’ and according to the results of structural studies, they have almost similar structures. Thus, presented results of magnetic measurements reflect the realistic behaviours of both nanostructures.

**Figure 6:**
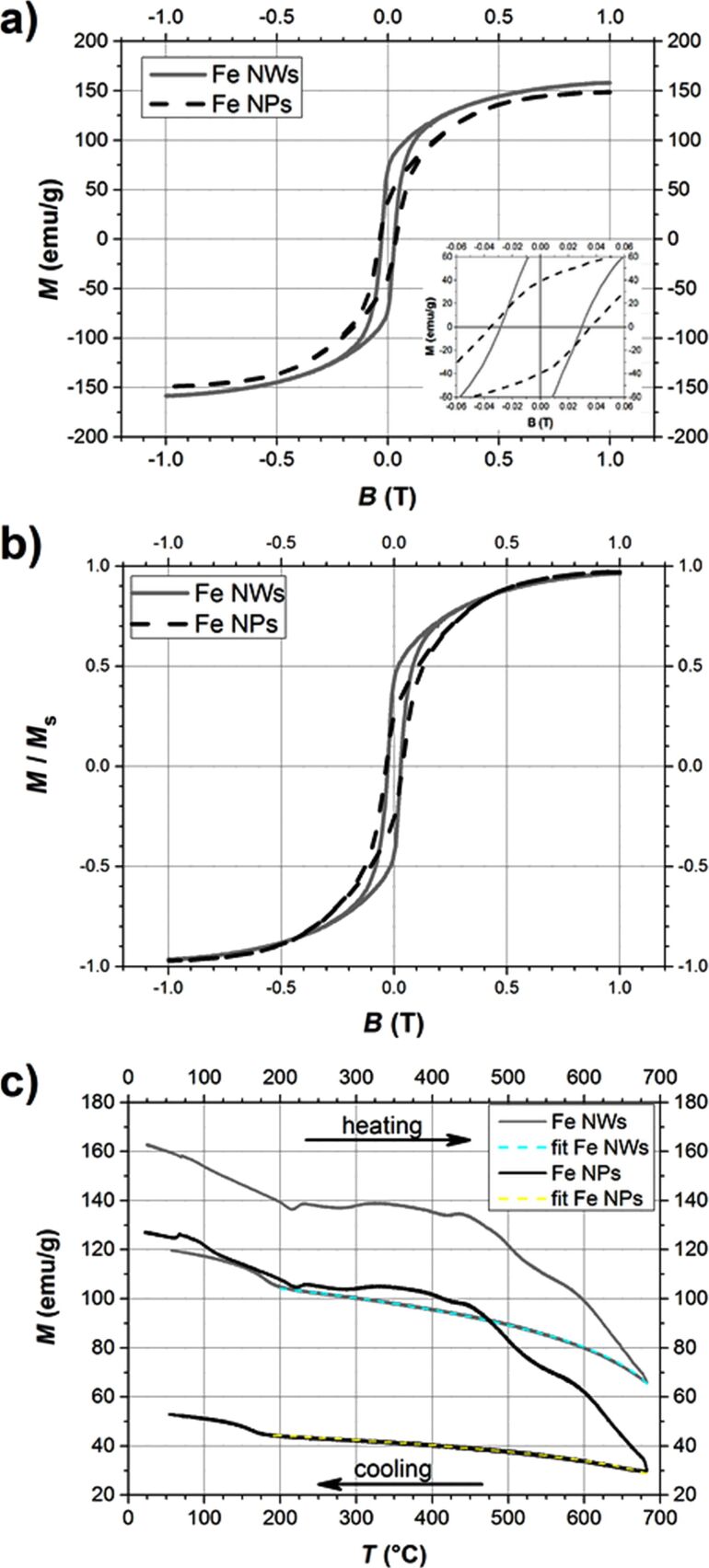
a) Magnetization hysteresis loops of iron nanowires and nanoparticles at room temperature (Inset – magnification of hysteresis); b) Normalized magnetization hysteresis loops of both studied nanostructures; c) Magnetization as a function of temperature curves recorded for iron nanowires and nanoparticles at magnetic field of 0.6 T (grey and black lines correspond to iron nanowires and iron nanoparticles, respectively).

Figure 6a and Figure 6b present the magnetization hysteresis of Fe NWs and Fe NPs, which have been registered at room temperature in the range of magnetic field from −1 T to 1 T, while Figure 6c shows the magnetization as a function of the temperature in the magnetic field of 0.6 T. The magnetic properties of iron nanowires and iron nanoparticles described by the values of coercivity (*H*_c_), saturation magnetization (*M*_s_), remanent magnetization (*M*_R_), squareness ratio (*M*_R_/*M*_s_) and Curie temperature (*T*_C_) are collected in Table 3.

**Table 3:** Magnetic properties of iron nanowires and iron nanoparticles; *H*_c_ – coercivity, *M*_s_ – saturation magnetization, *M*_R_ – remanent magnetization, *T*_C_ – Curie temperature.

	Fe NWs	Fe NPs

*H*_c_ (Oe)	300	360
*M*_s_ (emu/g)	164	153
*M*_R_ (emu/g)	70	39
*T*_C_ (°C)	722	747
*M*_R_/*M*_s_	0.43	0.25

In this work, the values of magnetic moments are given per unit of total mass (emu/g), so both investigated nanomaterials exhibit the magnetization of inward iron core and outward iron oxide layers as the whole structures. Therefore, the saturation magnetizations of iron nanowires as well as of nanoparticles do not reach the value of bulk iron (218 emu/g at 20 °C [9]). It can be seen that the values of *M*_s_ and *M*_R_/*M*_s_ of Fe NWs are higher than those of Fe NPs. Such observations have been already noted but they have concerned the nanomaterials with different ‘histories’ with regard to their fabrication processes or with different chemical compositions [10–11].

The saturation magnetization of iron nanowires and iron nanoparticles equals 164 emu/g and 153 emu/g, respectively. These values have been determined by plotting the measured magnetization of the high-field part of the *M*(*B*) curve versus *H*^−2^ and following extrapolation of magnetization to the infinite field limit (*H*^−2^ = 0). Furthermore, these values can be compared with the roughly estimated magnetizations considering the contribution of iron in the individual phases delivered from the Mössbauer studies and the saturation magnetizations of their bulk material equivalents. Assuming that at room temperature the saturation magnetization of crystalline α-Fe and amorphous iron are around 218 emu/g [9] and 130 emu/g [24], respectively, and knowing that amorphous iron oxides exhibit paramagnetic behaviour [20,22], and considering the magnetization of distorted iron oxides to be about the value of bulk magnetite (Fe_3_O_4_) of around 92 emu/g [25], the estimated magnetizations (*M*_cal._) of the investigated nanomaterials can be calculated by using the formula:

[2]
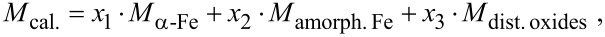


where *x*_1_ – percentage of crystalline α-Fe in the material, *M*_α-Fe_ – saturation magnetization of crystalline α-Fe, *x*_2_ – percentage of amorphous iron in the material, *M*_amorph. Fe_ – saturation magnetization of amorphous iron, *x*_3_ – percentage of distorted iron oxides in material, and *M*_dist.oxides_ – saturation magnetization of distorted iron oxides. The value of *M*_dist.oxides_ can be assumed as the saturation magnetization of Fe_3_O_4_ because our previous work has shown that an annealing of Fe NWs at 300 °C leads to the formation of oxide in the predominant form of magnetite [12]. This is also consistent with another report, in which the authors have demonstrated that with increasing core size of iron nanostructures the oxide shell is composed almost entirely of magnetite [26]. Applying Equation 2, the estimated magnetization of iron nanowires and iron nanoparticles was assumed to be around 150 emu/g and 147 emu/g, respectively. These values represent 91% and 96% of the experimental values of the saturation magnetization of Fe NWs and Fe NPs, respectively. The calculations of magnetization agree surprisingly well with the experimental results particularly since both studied nanomaterials exhibit complicated structures which include several iron-containing phases.

The coercivity and squareness ratio of iron nanowires and iron nanoparticles are similar and approach those found in the literature for the appropriate nanostructures with comparable sizes (see [2,27–28] for Fe NWs and [29] for Fe NPs). However, it can be noticed that the investigated nanoparticles exhibit a slightly higher coercivity and at the same time a lower *M*_R_/*M*_s_ ratio in comparison with nanowires (Figure 6b). As the chemical compositions of both studied nanostructures are almost identical, this observation is surprising at the first moment. In general, the effective anisotropy of the nanowires should be much higher due to the high uniaxial shape anisotropy, which is increased by the magnetocrystalline anisotropy with the easy axis oriented along the wire length caused by the application of the external magnetic field during the fabrication process. This indicates that during analysis of the hysteresis parameters it is also needed to consider the possible mechanisms of magnetization reversal besides the impact of simple anisotropy. According to the TEM measurements, the investigated iron nanowires can be treated as straight chains of iron nanoparticles. Therefore, the “chain-of-spheres model” can be applied for this nanomaterial [30]. Moreover, this model can be used only for the single-domain particles with diameters of around 100 nm [9], which matches perfectly with the average sizes of both studied nanomaterials. In single-domain systems, the magnetization reversal can occur via spin rotation [31] and according to the “chain-of-spheres model”, there are two possible mechanisms. One of them called the “parallel rotation mechanism” is related to the simultaneous parallel rotation of each dipole moment in the chain. In fact, this mechanism can be also used to describe the spin rotation in a single nanoparticle or in a group of randomly-oriented nanoparticles. The second mechanism, called the “fanning mechanism”, is associated with a fanning of magnetic moments along the chain that alternate in the sense of their rotation from one sphere to the next one [30]. Both mechanisms have been examined numerically for different length of nanoparticles chains [30,32]. They have been also used for describing experimental results [33–34]. According to theoretical calculations and experimental results shown in different publications, the ”fanning mechanism” yields lower coercivity values than the ”parallel rotation mechanism”. Therefore, it is likely that the studied iron nanowires exhibit a lower coercivity because the magnetization reversal follows the “fanning mechanism”, while the "parallel rotation mechanism" occurs in the iron nanoparticle clusters.

Curie temperatures of iron nanowires as well as of nanoparticles are estimated based on the measurements of magnetization as a function of the temperature (Figure 6c). The obtained plots for both nanostructures have exactly the same shapes. It proves that both materials are structurally similar. However, the shapes of the plots are not as typical as for pure iron. The shapes of the heating curves are a bit wavy in the range of 200 °C to almost 500 °C due to slow oxidation and the following phase transformations of iron oxides. It is well-known that iron nanomaterials in the presence of even small quantities of oxygen tend to be oxidized immediately. The increasing temperature leads to progressive oxidation of iron to Fe_3_O_4_ (large nanostructures) or γ-Fe_2_O_3_ (small nanostructures) and following transformation to α-Fe_2_O_3_ [12,15]. Above 550 °C, it seems that the studied nanowires as well as nanoparticles consist of crystalline α-Fe_2_O_3_ located on the surface of the investigated nanomaterials and an α-Fe core, which do not change the structural properties during further heating. The effects of iron oxidation and the following phase transformations of iron oxides have a greater impact on the decrease of magnetization in the case of iron nanoparticles. It is related to the fact that Fe NPs have a larger surface area exposed to the atmosphere than Fe NWs.

The ferromagnetic–paramagnetic transition was not measured directly because the applied VSM setup is equipped with a furnace, which allows one to heat the samples only up to about 950 K (ca. 677 °C). Thus, the Curie temperature for both studied nanomaterials is calculated applying a simple formula:

[3]
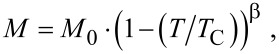


where *M* – calculated magnetization corresponding to changing temperature, *M*_0_ – fitted initial value of magnetization, *T* – changing temperature, *T*_C_ – fitted Curie temperature and β – fitted critical exponent. Equation 3 is associated with the theory of phase transitions occurring in the ferromagnets and uses a critical exponent, which can be found theoretically within the framework of different models describing the magnetic behaviour around the critical temperature (for instance the most common Heisenberg model). However, the applied formula allows for a very rough estimation of *T*_C_ and β originating from the extrapolation of fitted plots for both cooling curves. The fitting parameters determined by using Equation 3 are presented in Table 4. The estimated Curie temperatures of Fe NWs and Fe NPs are slightly lower than the Curie temperature of the bulk crystalline iron (770 °C [9]) and they could be different than the real *T*_C_ of studied nanostructures due to the application of a quite simple model fitted for the cooling curves as well as the calculation error. Additionally, these differences may be caused by certain inhomogeneities, admixtures and proximity effects of the iron core with respect to paramagnetic iron oxide phases that have been formed during the oxidation reaction [12,15] and have become paramagnetic at temperatures lower than ferromagnetic-paramagnetic transition of α-Fe. On the other hand, it is worth noting that the measurements of magnetization as a function of the temperature confirm that even though both fabricated nanomaterials change their structures during the heating, they still contain the crystalline iron, because none of iron-based oxides or hydroxides exhibit such high Curie temperature as pristine iron.

**Table 4:** Fitting parameters of the magnetic phase transition; *M*_0_ – fitted initial value of magnetization, β – fitted critical exponent, *T*_C_ – fitted Curie temperature.

	Fe NWs	Fe NPs

*M*_0_ (emu/g)	110.5	47.0
β	0.18	0.20
*T*_C_ (°C)	722	747

## Conclusion

In this work, possible structures of iron nanowires and iron nanoparticles have been determined, which indeed are very similar. Both nanomaterials reveal core–shell structures and are constructed of crystalline α-Fe cores, which are covered by a layered structure composed of: amorphous iron, amorphous iron oxides and distorted iron oxides (the layer order from core to surface). Additionally, iron nanowires are built from the self-assembled iron nanoparticles, which are separated by the very thin interface.

It is also shown that even if iron nanowires and iron nanoparticles have almost the same chemical compositions and similar dimensions, they always exhibit different magnetic properties. The only difference, which influences the samples, is the application of the external magnetic field during the fabrication process of iron nanowires. The external field enables the formation of wire-like nanomaterial, which actually should be considered as straight chains of single domain iron nanoparticles with dipolar interactions between them. These interactions result in the strong uniaxial shape anisotropy with the easy axis of magnetization parallel to the length of the wires. This stabilizes the magnetization distributions and causes that the squareness ratio (*M*_R_/*M*_s_) is higher in the studied nanowires. Besides that, the nanoparticles forming Fe NWs as well as the studied Fe NPs are in the regime of magnetic single-domains with regard to their dimensions. Therefore, the magnetization reversal process of iron nanowires can be described by the “chain-of-spheres model” with the “fanning mechanism”. This is the process with lower energy than the spin rotation occurring in the investigated iron nanoparticles, which can be associated with the “parallel rotation mechanism”. This is reflected in the lower coercivity value of the iron nanowires.

The estimated Curie temperatures of Fe NWs and Fe NPs are slightly lower than the Curie temperature of bulk crystalline iron. This can be mainly related to the interactions between the iron cores and the paramagnetic iron oxide phases, which have been formed during the oxidation reaction and have become paramagnetic at temperatures lower than ferromagnetic–paramagnetic transition of pure iron.

Moreover, presented studies confirm the previous reports [10–11] about the superiority of nanowires over nanoparticles regarding to their values of saturation magnetization.

## Experimental

**Fabrication of Fe NWs and Fe NPs:** Iron nanowires as well as iron nanoparticles have been prepared in a similar manner as described in [3,12,15,35–36]. Therefore, the fabrication procedures are described briefly here. Both nanostructures were synthesized through a chemical reduction of 0.2 mL of a 0.5 M aqueous solution of iron(III) chloride hexahydrate (FeCl_3_·6H_2_O; 98%, Sigma-Aldrich) with 2 mL of a 1 M aqueous solution of sodium borohydride (NaBH_4_; 98%, Sigma-Aldrich). This reaction was performed with an external magnetic field of about 0.2 T (2000 Gauss) to ensure the formation of a nanowires, or without an external magnetic field to get iron nanoparticles. Moreover, a constant argon flow was set to purge out the dissolved oxygen from the initial solution of the iron(III) precursor. Inert gas was present until all of the reducing agent (NaBH_4_) was added into the iron salt solution. Then, the obtained products were washed several times with ethanol (99.5%, Shimakyu’s Pure Chemicals Company) to remove the reaction residues and then they were dried in a vacuum oven at 80 °C.

**Characterization of investigated nanostructures:** The morphology of iron nanowires and iron nanoparticles were characterized by a transmission electron microscope (TEM JEOL JEM 3010). The element analysis was performed by a field-emission scanning electron microscopy (FE-SEM Hitachi SU8020) with an energy dispersive X-ray spectrometer (EMAX Evolution X-Max). The structural properties of both synthesized samples were characterized at room temperature by a Bruker D2 Phaser diffractometer (XRD) equipped with a Cu sealed tube (X-ray source; λ = 0.1542 nm) and a LYNXEYE detector and also a standard Mössbauer spectrometer (POLON) equipped with a ^57^Co/Rh source of γ-radiation placed on a vibrator, working in a constant acceleration mode. Magnetic properties were determined by means of an Oxford Instruments Ltd. vibrating sample magnetometer (VSM) equipped with a furnace which allowed to heat the samples up to around 950 K (ca. 677 °C). The samples were heated with a rate of 5 °C/min.
